# A Unique G-Quadruplex Aptamer: A Novel Approach for Cancer Cell Recognition, Cell Membrane Visualization, and RSV Infection Detection

**DOI:** 10.3390/ijms241814344

**Published:** 2023-09-20

**Authors:** Chao-Da Xiao, Ming-Qing Zhong, Yue Gao, Zheng-Lin Yang, Meng-Hao Jia, Xiao-Hui Hu, Yan Xu, Xiang-Chun Shen

**Affiliations:** 1 State Key Laboratory of Functions and Applications of Medicinal Plants, School of Pharmaceutical Sciences, Guizhou Medical University, Guiyang 550025, China; zhongmingqing163@163.com (M.-Q.Z.); gaoyue2308@163.com (Y.G.); crazyyang168@163.com (Z.-L.Y.); jiamenghao0522@163.com (M.-H.J.); 19985602747@163.com (X.-H.H.); 2 The Key Laboratory of Optimal Utilization of Natural Medicine Resources, School of Pharmaceutical Sciences, Guizhou Medical University, Guiyang 550025, China; 3 Division of Chemistry, Department of Medical Sciences, Faculty of Medicine, University of Miyazaki, Miyazaki 889-1692, Japan; xuyan@med.miyazaki-u.ac.jp

**Keywords:** G-quadruplex, nucleolin, cancer cell identification, detection of RSV infection, cell surface staining

## Abstract

Surface staining has emerged as a rapid technique for applying external stains to trace cellular identities in diverse populations. In this study, we developed a distinctive aptamer with selective binding to cell surface nucleolin (NCL), bypassing cytoplasmic internalization. Conjugation of the aptamer with a FAM group facilitated NCL visualization on live cell surfaces with laser confocal microscopy. To validate the aptamer-NCL interaction, we employed various methods, including the surface plasmon resonance, IHC-based flow cytometry, and electrophoretic mobility shift assay. The G-quadruplex formations created by aptamers were confirmed with a nuclear magnetic resonance and an electrophoretic mobility shift assay utilizing BG4, a G-quadruplex-specific antibody. Furthermore, the aptamer exhibited discriminatory potential in distinguishing between cancerous and normal cells using flow cytometry. Notably, it functioned as a dynamic probe, allowing real-time monitoring of heightened NCL expression triggered by a respiratory syncytial virus (RSV) on normal cell surfaces. This effect was subsequently counteracted with dsRNA transfection and suppressed the NCL expression; thus, emphasizing the dynamic attributes of the probe. These collective findings highlight the robust versatility of our aptamer as a powerful tool for imaging cell surfaces, holding promising implications for cancer cell identification and the detection of RSV infections.

## 1. Introduction

Nucleolin (NCL) has been shown to be involved in several important biological processes, such as ribosome biogenesis [1,2], regulation of DNA replication and repair [3,4,5], gene expression regulation [6,7], and apoptosis [8,9]. Typically, more than 90% of NCL is localized within the nucleolus in normal cells [10]. Nevertheless, various physiological and pathological processes can cause changes in the subcellular distribution of NCL. For example, in cancerous cells, NCL can be significantly overexpressed on the cellular membrane [10,11,12,13]. Recent research has also indicated that cell surface NCL is even tumor-specific, both in vitro and in vivo [14]. Several studies have demonstrated that overexpressed NCL on the membrane surface of cancer cells is a key target that interacts with multiple proteins and is associated with cancer cell proliferation and migration [15]. Moreover, some research suggests that NCL on the cancer cell membrane surface regulates cell adhesion and metastasis [16,17]. Both of these findings indicate that NCL on the cell surface could serve as a potential target for cancer treatment or diagnosis. 

There is also mounting evidence that NCL translocated on the cell surface plays a crucial role in viral reception. Several well-known viruses, including HIV (human immunodeficiency virus), RSV (respiratory syncytial virus), HPIV-3 (human parainfluenza virus type 3), IAV (various influenza A viruses), EVA71 (enterovirus A 7), and CVB (B type coxsackieviruses), have been shown to interact with NCL on the cell surface and regulate the attachment and entry process of the virus [18]. Among the mentioned viruses, RSV has been extensively studied due to its unique mechanism of infection. The virus binds and enters host cells through RSV-G and RSV-F envelope proteins. When the RSV-F glycoprotein binds to IGFR-1, it activates PKCζ, resulting in the recruitment of NCL from the cell nucleus to the membrane surface. Then, NCL plays a crucial role in facilitating the fusion of viral and host cell membranes, enabling RSV replication to occur [19,20]. This process can be inhibited by anti-NCL antibodies and the nucleic acid aptamer AS1411, highlighting the importance of monitoring changes in NCL on the cell surface to understand the mechanism of RSV infection or to detect the infection of RSV [21,22].

Nucleic acid aptamers exhibit high affinity for specific targets [23] and possess several unique advantages, including low synthesis cost, easy modification, small molecular weight, good solubility, and negligible immune responses. These properties make them highly promising for a variety of applications in the fields of diagnosis and anti-tumor therapy [24]. For instance, the extensively researched aptamer AS1411 exhibits high binding affinity towards NCL and has demonstrated anti-proliferative properties against a variety of cancer cells [25,26]. AS1411 binds to NCL on the surface of cancer cells and is internalized, exhibiting selective cytotoxicity against cancer cells. Upon conducting further structural analysis, it is believed that the binding between AS1411 and NCL is dependent on the formation of a G-quadruplex structure by the aptamer’s sequence [27,28,29,30]. Furthermore, the other G-quadruplex forming aptamers also showed high affinity to NCL [31,32]. 

G-quadruplexes (G4s) are intricate four-stranded structures that can be formed by DNA/RNA sequences that are rich in guanine (G) residues [33,34,35]. Compared to unstructured sequences, G-quadruplex-forming aptamers exhibit enhanced thermodynamic and chemical stability, and are resistant to a variety of serum nucleases [36,37]. Thus, there is growing interest in developing NCL-specific aptamers that can form a G-quadruplex. In this study, we presented a novel aptamer (referred to as Apt-3) that adopted a G-quadruplex structure and specifically targeted the NCL on the surface of cancer cells without being internalized into the cytoplasm. We further labelled Apt-3 with a FAM group at the 5’-end (referred to as Apt-3-F) and observed that it could selectively stain the cancer cell membrane which enabled us to distinguish cancer cells from normal ones. Additionally, we were able to track the increasing expression of NCL on the membrane of normal cells induced by RSV. 

In summary, we introduced a simple and effective method for imaging the NCL protein on the cell surface using an aptamer-based dye, which only required co-culture and wash procedures without the need for fixation. This approach offered significant potential for advancing the research in cell biology and disease diagnostics, providing a promising alternative to the complex genetic or metabolic manipulations required by most current techniques for labelling cell membrane proteins [38]. 

## 2. Results 

### 2.1. Finding the Aptamer Binding to NCL on Cell Surface

**Figure 1:**
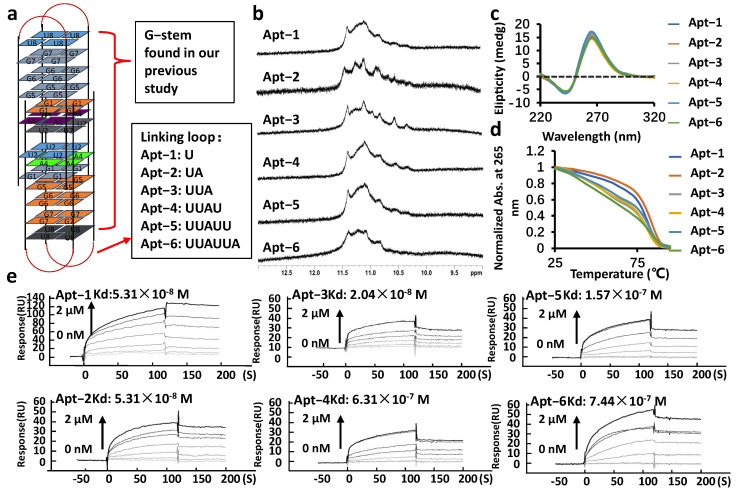
(**a**) The aptamer design concept in this study. The G-stem identified in our previous study is denoted by red parentheses. The loop with varying content is depicted within a black box indicated by red arrow. (**b**) Imino peaks of different aptamers. (**c**) CD spectrum and (**d**) melting curves of aptamers. (**e**) Sensorgrams showing aptamer-NCL binding (resonance units vs. time) with increasing aptamer concentration from 0 μM to 2 μM, indicated by darker curves.

The G4s consistently displayed a strong affinity towards NCL [31]. Given that G-rich telomeric RNA sequences are capable of forming G4 structures and that the loop length can significantly impact the binding of G4 to NCL, we designed G4 structures featuring the telomeric RNA core identified as forming a G4 structure in our prior research, with varying linking loop lengths. (Table 1, Figure 1a) [31,33]. Utilizing nuclear magnetic resonance (NMR) experiments, we observed clear imino peaks ranging from 10 to 12 ppm, indicating that all the sequences used in this study formed G-quadruplexes in a K^+^ solution (Figure 1b). Furthermore, all sequences exhibited a positive peak at 265 nm and a negative peak at 240 nm in the CD spectrum, indicating that all aptamers adopted a parallel G4 structure (Figure 1c) [39,40,41,42]. The melting curves indicated that all G4s were relatively thermally stable, with Tm values higher than 70 °C (Figure 1d). The greater stability observed in Apts in comparison to the extensively studied aptamer AS1411, which exhibits a melting temperature (Tm) value of 50 °C [30], suggests a higher potential for Apts to form G-quadruplex structures capable of binding to NCL. This enhanced stability may make Apts promising candidates for future applications. 

Subsequently, we utilized surface plasmon resonance (SPR) experiments to determine the binding affinity of aptamers towards NCL. Results from these experiments revealed that all aptamers that can bind to NCL and Apt-3 displayed the strongest binding affinity towards NCL with a *K_d_* value of 2.04 × 10^−8^ M (Figure 1e). In contrast, the mutant sequence unable to form a G4 structure showed no binding to NCL (Figure S1). Therefore, the binding of Apt-3 to NCL is dependent on its G4 structure. These findings collectively led us to choose Apt-3 for further investigation. NCL plays crucial roles in both the nucleus and cytoplasm. Consequently, molecules such as aptamers, peptides, or pseudopeptides targeting NCL have consistently demonstrated cytotoxic effects [10,12]. Consequently, we investigated the cytotoxicity of Apt-3 on a variety of cancer cell lines, including the MCF7, Hela, and MDA MB 468. Surprisingly, our results indicated that, for cancer cells, the cytotoxicity of Apt-3 is much lower than that of AS1411, with an average *CC_50_* value of Apt-3 being at least 10 times higher than AS1411 (Table 2, Figure S2). Thus, that result made us wonder whether Apt-3 can truly bind to NCL in the cell. Regarding aptamers such as the AS1411, their cytotoxicity mechanism involves an initial attachment to NCL on the cell surface, followed by cellular uptake through a prevalent form of endocytosis in cancer cells. Subsequently, it disrupts NCL-mediated intracellular signaling and results in cytotoxicity [27,43,44]. Therefore, we tested whether Apt-3 can bind to NCL on the cell surface. To investigate Apt-3’s ability to bind to NCL on the cell surface, we performed immunohistochemistry (IHC) using the NCL antibody to detect NCL on the cell surface and utilized Apt-3 as a competitor for the NCL antibody. Flow cytometry analysis revealed that Apt-3 effectively competed with the antibody for the binding to NCL on the cell surface. Conversely, the mutant sequence demonstrated no notable impact in the interaction between NCL and its corresponding antibody. (Figure S3). These findings provided further evidence that Apt-3 had the ability to bind to NCL on the cell surface. Importantly, the data suggest that Apt-3 may be cell membrane-anchorable and not internalized into the cytoplasm, thereby avoiding interference with NCL function within the cell and minimizing its potential cytotoxicity. These results encourage further investigation of Apt-3 as a potential tool for detecting NCL on the cell surface.

### 2.2. Detecting the NCL on Cell Surface by Apt-3-F 

**Figure 2:**
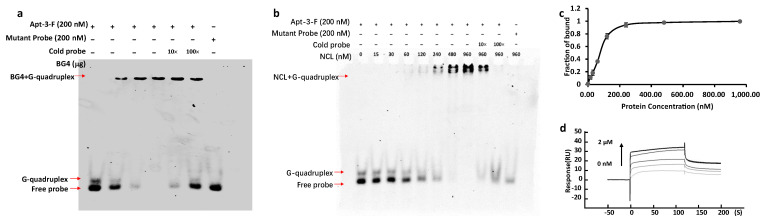
(**a**) EMSA assays using BG4 to verify the G4 structure formed by Apt-3-F fragment. (**b**) EMSA analysis showed that recombinant NCL was selectively bound to the G4 structure formed by Apt-3-F. The cold probe was the unlabeled Apt-3 probe. (**c**) Binding curves of NCL to Apt-3-F, in which the binding fraction data were obtained from EMSA experiments. The *K_d_* values were obtained by fitting the mobility shift data to the equation given in Section 4. (**d**) Sensorgrams (resonance units vs. time) of SPR experiments depicting Apt-3-F binding to NCL.

To enable Apt-3 to be used for staining, we added a FAM group to the 5’-end of the aptamer (designated Apt-3-F). We conducted the electrophoretic mobility shift assay (EMSA) utilizing the BG4 antibody to validate the structure formed by Apt-3-F. Illustrated in Figure 2a, the findings unequivocally demonstrated the interaction between Apt-3-F and BG4, further corroborating the formation of the G-quadruplex (G4) structure by Apt-3-F. EMSA was also employed to ascertain the binding between Apt-3-F and NCL. As depicted in Figure 2b, the gel image distinctly exhibited a slower migration band corresponding to the complex of the NCL protein and Apt-3-F treated with K^+^. Notably, when a mutant probe was employed, wherein several Gs were substituted with Ts, the resultant sequence lost its capacity to form G4 structures, thereby yielding no observable slower migration band on the gel. This outcome strongly implies that NCL specifically recognizes the G4 structure formed by Apt-3-F. Upon analysis of the *K_d_* value, the determined value of Apt-3-F’s affinity to NCL was measured at 7.713 × 10^−8^ M, akin to the affinity value of Apt-3, as shown in Figure 2c. Complementary to these findings, surface plasmon resonance (SPR) analysis was also utilized to elucidate the binding dynamics between Apt-3-F and NCL. Depicted in Figure 2d, the results consistently exhibited that the inclusion of the FAM group insignificantly impacted the binding affinity of Apt-3 to NCL, with a *K_d_* value of 2.491 × 10^−8^ M.

**Figure 3:**
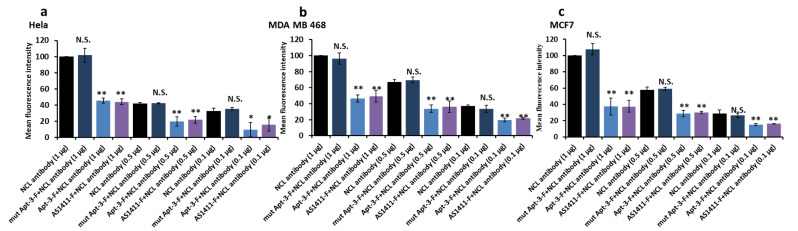
HC + flow cytometry confirmed Apt-3-F binding to NCL on cell membranes of Hela (**a**), MDA MB 468 (**b**), and MCF7 (**c**). Data were expressed as the mean ± SEM (*n* = 3). ** *p* < 0.01. * *p* < 0.05. (Student’s *t* test).

In addition, we utilized IHC in combination with flow cytometry to detect the binding of Apt-3-F to NCL on the cell surface. The results indicated that Apt-3-F can compete with the NCL antibody on the cell membrane to the same extent as Apt-3. Importantly, the mutant sequence incapable of forming the G-quadruplex structure failed to inhibit the binding between NCL and the antibody. This additional evidence further reinforces its prospective utility as a valuable tool for detecting NCL presence on the cell surface (Figure 3a–c). 

### 2.3. Cell Membrane Staining and Cancer Cell Indication by Apt-3-F

**Figure 4:**
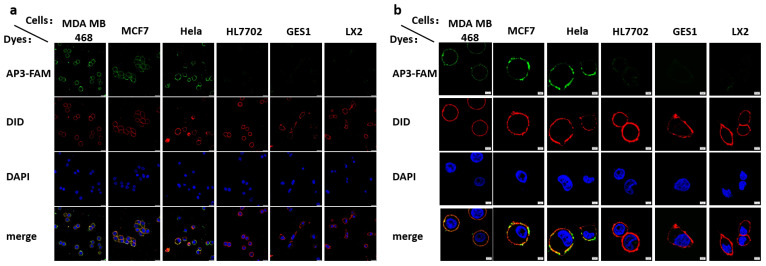
(**a**) Laser confocal microscope images of cancer (MDA MB 468, MCF7, Hela) and normal cells (HL7702, GES1, LX2) treated with 1 μM Apt-3-F. Scale bar: 20 μm. Green channel: *λex* = 488 nm, *λem* = 500–530 nm. Blue channel: *λex* = 405 nm, *λem* = 440–470 nm. Red channel: *λex* = 561 nm, *λem* = 600–700 nm. (**b**) High magnification images displaying the cell membrane staining of Apt-3-F. Scale bar: 5 μm.

Based on these findings, we employed Apt-3-F for staining the cell membrane in cancer cells by simply mixing it with the cells, followed by PBS washing. Then, we compared the staining results with those of normal cells. The laser confocal microscope image revealed that a vast majority of the fluorescence signals were located on the cell membrane in cancer cells, whereas a signal was barely observed in normal cells (Figure 4a,b). 

**Figure 5:**
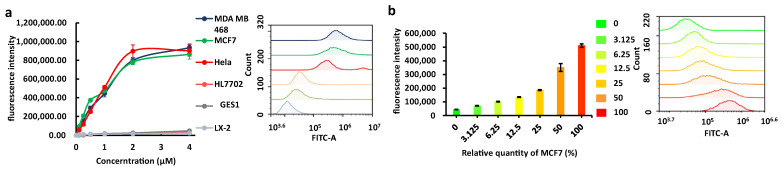
(**a**) Left side: mean fluorescence intensity of different cell staining with increasing concentrations of Apt-3-F detected by a flow cytometer (fixed wavelength at *λex* = 488 nm, *λem* = 530 nm). Right side: The flow cytometry representation results for various cells at a 1 μM concentration of Apt-3-F. (**b**) Fluorescence intensity of cancer cells (MCF7) and normal cells (LX2) at different staining ratios with a 1 μM concentration of Apt-3-F. Left side: The flow cytometry histogram results (fixed wavelength at *λex* = 488 nm, *λem* = 530 nm).

Moreover, the data obtained from flow cytometry experiments suggest that the intensity of fluorescence staining on cells is dependent on the concentration of Apt-3-F. Notably, at the same concentration of Apt-3-F, cancer cells exhibited fluorescence intensity levels up to 40 times higher than those observed in normal cells (Figure 5a). Furthermore, the fluorescence intensity of cells stained with Apt-3-F was positively correlated with the proportion of cancer cells in the cell population (Figure 5b). These results suggest that Apt-3-F can be utilized for specific staining of cancer cell membranes, enabling the distinction between cancer cells and normal cells. As a control, AS1411, known to internalize into the cytoplasm, was labelled with the FAM group at the 5’-end, generating AS1411-F that exhibited a fluorescence signal in the cytoplasm. Furthermore, AS1411-F displayed lower selectivity between cancer cells and normal cells when compared to Apt-3-F (Figure S4). Prior research has established that cytoplasmic NCL binds to the 3′-UTR of BCL2 mRNA, effectively protecting it from degradation. NCL plays a pivotal role in stabilizing BCL-2 mRNA aptamers’ function by binding to NCL, disrupting its functionality, and consequently repressing the expression of the BCL-2 protein in cells [44,45]. Thus, we proceeded to investigate whether Apt-3-F can interfere with the expression of BCL-2. The Western blot results showed that Apt-3-F did not significantly alter the expression of the BCL-2 protein. However, AS1411-F, as previously reported, entered the cytoplasm, and suppressed the expression of BCL-2 [43] (Figure S5). All together, these findings suggest that Apt-3-F may serve as a valuable tool for anchoring on the cell surface, detecting NCL, and differentiating between cancer and normal cells. 

### 2.4. RSV Infection Indicated by Apt-3-F 

**Figure 6:**
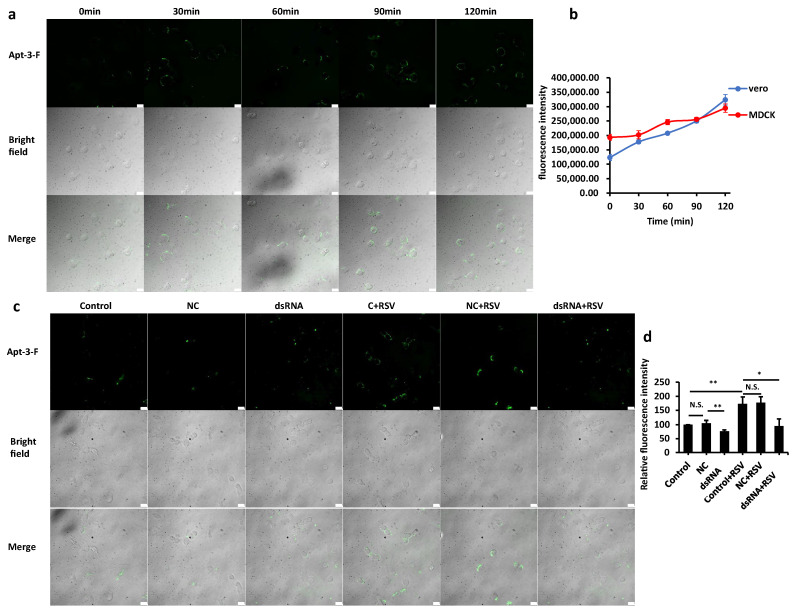
(**a**) Staining images of Vero cells treated with varying RSV infection times. Scale bar: 20 μm. (**b**) Mean fluorescence intensity variations of cells stained with increasing RSV infection time using 1 μM Apt-3-F, measured via flow cytometry. (**c**) Apt-3-F staining images of Vero cells treated with RSV and NCL dsRNA, Scale bar: 20 μm. (**d**) Fluorescence intensity detected by flow cytometry of Vero cells treated with RSV and NCL-dsRNA. Data were expressed as the mean ± SEM (*n* = 3). ** *p* < 0.01. * *p* < 0.05. N.S. (nonsignificant) = *p* > 0.05, vs. Ctrl group (Student’s *t* test).

To further validate the capability of Apt-3-F in detecting NCL on the cell surface, we examined the fluorescence signal on normal cells treated with RSV, which is known to induce the translocation of NCL from the nucleus to the cell surface. As depicted in Figure 6a,b and Figure S6, Vero cells and MDCK cells stained by Apt-3-F initially showed very low fluorescence signals, but the signals gradually increased with increasing RSV infection time. Flow cytometry analysis revealed a significant increase in the fluorescence signal of cell staining by Apt-3-F following RSV induction, with a 1.5-fold increase in MDCK cells and a 2.6-fold increase in Vero cells. Notably, this increase in the fluorescence signal was effectively disrupted when dsRNA was transfected into Vero cells, leading to suppression of NCL expression, as shown in Figure 6c,d and Figure S7. These compelling results provide additional evidence supporting the robust capability of Apt-3-F in accurately detecting NCL on the cell surface and the ability for indicating the cell infection of RSV.

## 3. Discussion

Surface staining has become an increasingly popular method for quickly applying exogenous stains to track cellular identity within mixed populations. Protein-specific antibodies were a significant development in allowing for the first rationally designed plasma membrane-specific dyes. However, the quality and efficiency of commercially available antibodies can vary dramatically, making it difficult to find suitable probes for reliable and reproducible results [46]. Monoclonal antibodies are also more expensive, and some antibodies require fixation for epitope recognition, which can limit their application for cell surface staining. Additionally, permeabilization can lead to unspecific internal staining, further limiting the utility of antibodies for this purpose. Here, the Apt-3-F nucleic acid aptamer can be used to stain cell surfaces with a simple staining procedure that does not require fixation. Additionally, the cost of producing stable-quality nucleic acid is much cheaper than producing monoclonal antibodies, making Apt-3-F a valuable alternative for cell surface staining. Furthermore, considering the demonstrated high stability in vitro, it is reasonable to speculate that such an aptamer may also exhibit substantial stability in the complex environment of biological tissues, further enhancing its potential for various applications.

**Figure 7:**
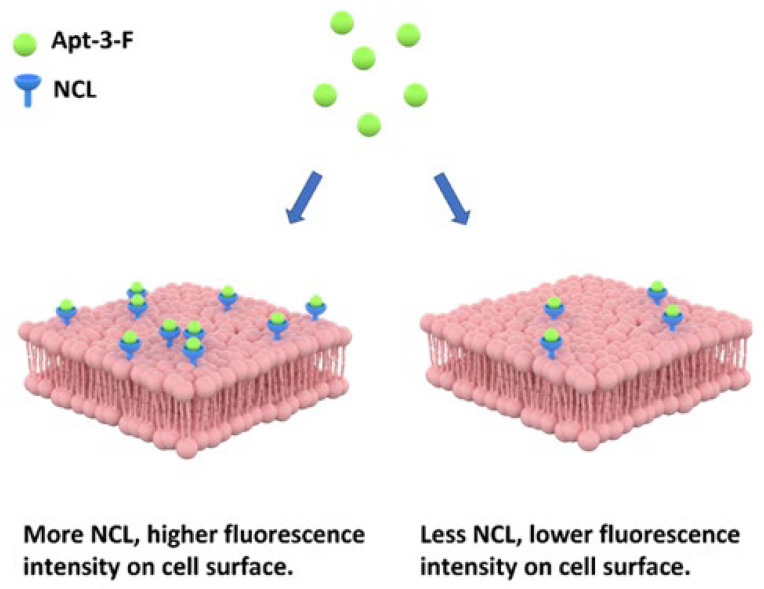
Schematic illustration depicting a membrane-anchored aptamer for monitoring NCL on the surface of live cells.

The G4 aptamers previously reported have been shown to internalize into the cytoplasm and exhibit cytotoxicity [47]. However, the uptake of these aptamers relies on the endocytosis process of the target cells. Various factors can affect endocytosis, including the size and shape of the particles. In the case of Apt-3, the non-denature gel image revealed that the 19-nt sequence exhibited lower mobility compared to the dT24 marker, indicating the potential formation of a unique intermolecular G4 structure (Figure S8). This distinct structure could potentially explain the differential uptake of Apt-3 compared to other G4s, as it remains anchored on the cell surface (Figure 7). Further structural analysis may contribute to understanding the mechanism underlying the uptake of Apt-3.

Traditionally, the SELEX (systematic evolution of ligands by exponential enrichment) technique has been employed for aptamer selection [48]. However, it has limitations: it can successfully isolate aptamers that bind to a particular target and it does not inherently prioritize the selection of aptamers with specific characteristics beyond target binding. Here, Apt-3-F exhibits the unique property of binding exclusively to NCL on the cell surface without internalizing into the cytoplasm. This property significantly enriches the landscape of aptamer development. Furthermore, delving deeply into the interaction between the conformation of the G-quadruplex structure formed by Apt-3-F and its internalization properties could provide valuable insights that can be applied to the design of aptamers.

## 4. Materials and Methods

### 4.1. Oligonucleotide Synthesis and Sample Preparation

The oligonucleotides were synthesized at 1 μmol scale using standard phosphonamidite solid-phase chemistry on an automatic DNA/RNA synthesizer (Nihon Techno Service, Kashima, Japan). After deprotection and detachment from the support, reverse high-performance liquid chromatography (Shimadzu Co., Suzhou, China) was used for purification, followed by desalting with a NAP^TM^-10 column (GE Healthcare, Chicago, IL, USA). Two buffer solutions were used for preparing the aptamers: Apt-3 in 100 mM KCl and 10 mM K_3_PO_4_ (pH 7.0), and AS1411 in 100 mM NaCl and 10 mM Tris-HCl (pH 7.0). The samples were heated to 95 °C for 5 min, gradually cooled to room temperature, and incubated overnight at 4 °C. Oligonucleotides are labeled at the 5’-end using 6-FAM groups via an amide bond linker. The RNA oligonucleotides used in this study are in-house synthesized.

### 4.2. Circular Dichroism (CD) Spectroscopy Experiments

The CD spectrum of Apt-3 was obtained using a Chirascan spectrophotometer (Applied Photophysics, Surry, UK). Apt-3 at a concentration of 10 μM was denatured at 95 °C for 5 min and incubated overnight at 4 °C in a 400 μL solution containing 100 mM KCl and 10 mM K_3_PO_4_ buffer (pH 7.0). The CD spectrum was recorded in a quartz cuvette at 25 °C using a wavelength range of 220–320 nm and a scanning speed of 100 nm/min. The melting curve was obtained by heating the sample from 25 °C to 95 °C at a rate of 1 °C/min, and the Tm was determined as the temperature at which the CD signal value decreased by half.

### 4.3. Surface Plasmon Resonance (SPR) Experiments 

The SPR experiments were performed on Biacore 3000 (Cytiva, Logan, UT, USA) by immobilizing purified recombinant human NCL (NM_005381) on a CM5 chip via amine coupling. The binding analysis was conducted in a HEPES-KCl buffer (0.01 M HEPES, 0.15 M KCl, 3 mM EDTA, pH 7.4) at a flow rate of 30 μL/min with association and dissociation times of 120 s and 180 s, respectively. Aptamers were annealed and diluted in a HEPES-KCl buffer. The analytes with concentrations ranging from 0 nM to 2 μM were injected sequentially, and the surface of the chip was regenerated with 1 M KCl. The response curves of each concentration were recorded and analyzed using instrument software Trace Drawer 1.6.1.

### 4.4. Cell Culture and Cell Viability Assays

We obtained MDA MB 468, MCF7, HL7702 Vero and MDCK cells from ATCC (MA, Rockefeller, MD, USA), and Hela and LX2 cells from the Kunming cell bank of the Chinese Academy of Sciences. GES1 cells were purchased from BNCC. All cells were grown in DMEM supplemented with 10% FBS, 100 U/mL penicillin, and 100 μg/mL streptomycin at 37 °C in a 5% CO_2_ incubator.

Cells were seeded at a density of 2 × 10^3^ in 96-well plates and treated with varying concentrations of AS1411 (0–32 μM) or Apt-3 (0–128 μM) for 5 days. MTT assay was performed by adding 15 μL of MTT (5 mg/mL) and incubating cells for 4–6 h. The formazan was dissolved in 150 μL of DMSO, and the absorbance was measured at 490 nm using a spectrophotometer (Bio Tek, Winooski, VT, USA). The experiment was repeated three times.

### 4.5. Electrophoretic Mobility Shift Assay (EMSA) 

The wild-type and mutant Apt-3 sequences were tagged with a FAM label at their 5’-ends. Subsequently, the probe was dissolved in a solution containing 10 mM K_3_PO_4_ buffer (pH 7.0) and 100 mM KCl. The solution was subjected to a temperature of 95 °C for 5 min, followed by a gradual cooling to room temperature to facilitate the formation of a G-quadruplex structure. The binding process occurred within a mixed system comprising a 5× binding buffer (40 mM Tris, 30 mM KCl, 1 mM MgCl_2_, 1 mM DTT, pH 8.0), the probe (200 nM), and varying concentrations of NCL protein or the BG4 antibody (MABE917, Sigma–Aldrich, St. Louis, MO, USA). This mixture was allowed to interact at room temperature for a duration of 25 min. In the case of a competitive reaction, additional unlabeled cold probes were introduced to the mixture. Subsequently, the reaction products were combined with 30% glycerol (*v*/*v*) and loaded into a 12% non-denaturing polyacrylamide gel. Electrophoresis was carried out at 80 V for 2 h at 4 °C using a 1× TBE buffer (Tris-Boric 0.045 M, EDTA 0.001 M, pH 8.0) containing 20 mM KCl. The resulting image was captured using a UV transilluminator integrated into the ChemiDoc XRS System developed by Bio-Rad Laboratories. The data obtained from the image were subsequently analyzed utilizing the Image Lab 5.2 software.

Lastly, the dissociation constant (*K_d_*) was determined by identifying the concentration of protein at which half of the free probe was bound. Firstly, dataset was presented as a graph depicting the relationship between the proportion of binding probes to total probes (Y) and protein concentration (X). Nonlinear regression analysis was performed using Prism 9.0.0 software to calculate the *K_d_* using the equation: Y = X/{*K_d_* + X}. 

### 4.6. Immunohistochemistry-Based (IHC-Based) Flow Cytometry for Assessing Aptamer Binding to Cell Surface Nucleolin

Aptamers and cell surface nucleolin binding assays were conducted followed the reported method using flow cytometry [22] (ACEA Biosciences, San Diego, CA, USA). MDA MB 468, MCF7, and Hela cells were collected with 2 mM EDTA and incubated with aptamers (2 μM) at 4 °C for 30 min. After being washed with phosphate-buffered saline (PBS), cells were incubated with different concentrations of anti-Nucleolin primary antibody (Ab22758, Abcam, Boston, MA, USA) for 1 h at 4 °C, followed by CoraLite647-conjugated Anti-Rabbit IgG secondary antibody (5 µg/mL) for Apt-3-F and AS1411-F, or Aleax Fluor 488-conjugated Anti-Rabbit IgG secondary antibody (5 µg/mL) for Apt-3 and AS1411. The fluorescence intensity was measured by flow cytometry and analyzed using Novoexpress 1.5.6 software^®^. The experiment was repeated three times.

### 4.7. Laser Confocal Imaging

Images of cancer cells and normal cells stained with Apt-3-F or AS1411-F (1 μM) were acquired using an Olympus VS200 confocal microscope. The cells were seeded into 6-well plates and incubated for 12–16 h. Subsequently, Apt-3-F or AS1411-F was added to the cells, and they were washed 3 times with PBS. The samples were then fixed with 4% paraformaldehyde and stained with DAPI (10 μg/mL) for nuclei and DID (10 μg/mL) for the cell membrane. Confocal microscopy with excitation wavelengths of 405 nm for DAPI, 488 nm for FAM, and 560 nm for DID were used to obtain the images.

To monitor changes in NCL expression on the cell surface induced by RSV infection, MDCK or Vero cells were seeded into 6-well plates with a cover glass and incubated for 1 h. Cells were exposed to RSV and then incubated for different periods of time in the incubator. At indicated time points, Apt-3-F or AS1411-F (1 μM) was added, incubated for 1 h at 37 °C, and washed with PBS three times. The images were visualized by confocal microscopy (Olympus, Tokyo, Japan) after the slides were sealed with an anti-fluorescence quenching reagent.

### 4.8. Flow Cytometry Analysis for Aptamer Staining in Cells

To analyze the staining of cancer cells by Apt-3-F, cells (2 × 10^5^) were seeded into 6-well plates and treated with Apt-3-F or AS1411-F (600 μL) at varying concentrations for 12–16 h at 37 °C. After removing residual liquid with PBS, the cells were treated with an additional 1 mL of PBS at 37 °C for 3 min to remove non-specific binding. The detached cells were then analyzed for fluorescence intensities using a flow cytometer.

To analyze changes in NCL expression on the cell surface induced by RSV infection, MDCK or Vero cells (2 × 10^5^) were infected with RSV (MOI = 0.01) for 1 h and incubated in a growth medium with 2% FBS for different periods. At specific time points, cells were stained with Apt-3-F, washed with PBS, and analyzed for fluorescence intensity using a flow cytometer.

### 4.9. Western Blotting

The BCL-2 protein expression level was detected in the cells using Western blotting after incubating Hela cells (2 × 10^5^) with Apt-3-F or AS1411-F (10 μM) for 48 h. Proteins were quantified, denatured, electrophoresed, and transferred onto an FVDF membrane. This membrane was blocked with 5% skimmed milk, incubated with anti-β actin (Proteintech, 66009-1, Wuhan, China) or anti-BCL-2 (Proteintech, 26593-1), and then incubated with the second antibody HRP-conjugated Affinipure Goat Anti-Mouse IgG(H + L) (SA00001-1, Proteintech) or HRP-conjugated Affinipure Goat Anti-Rabbit IgG(H + L) (SA00001-2, Proteintech). Finally, the membrane was incubated with highly sensitive ECL (Enhanced Chemiluminescent, New Cell & Molecular Biotech Co., Ltd. Suzhou, China), and blots were scanned on a ChemiDoc™ XRS system (BIORAD, Hercules, CA, USA) and analyzed with Image Lab (BIORAD). 

### 4.10. Nuclear Magnetic Resonance (NMR) Experiments 

The aptamers’ ^1^H NMR spectrum was acquired using a 600 MHz nuclear magnetic resonance spectrometer (Bruker, Karlsruhe, Germany). The samples, with a concentration of 1 mM, were dissolved in a 150 μL solution consisting of 90% H_2_O and 10% D_2_O, and were supplemented with 100 mM KCl and a 10 mM K_3_PO_4_ buffer at pH 7.0. The NMR spectra were recorded at a temperature of 25 °C.

### 4.11. RNA Interference (RNAi)

DsRNA specific for the targeting the NCL gene was synthesized by GenePharma (Shanghai, China). The designed dsRNA sequences were as follows: NCL sense: 5′-CGGCUUUCAAUCUCUUUGUTT-3′ and NCL anti-sense: 5′-ACAAAGAGAUUGAAAGCCGTT-3′. For the negative control (NC), the dsRNA sequences used were sense: 5′-UUCUCCGAACGUGUCACGUTT-3′ and anti-sense: 5′-ACGUGACACGUUCGGAGAATT-3′.

Vero cells were seeded in 6-well plates and incubated for 16–24 h in an incubator. Following the manufacturer’s instructions, cells were transfected with 2 μg of either NCL dsRNA or negative control dsRNA using Lipofectamine 2000 reagent (Invitrogen, Waltham, CA, USA). After 36 h, gene silencing was evaluated by Western blotting.

## 5. Conclusions

In summary, we developed a unique aptamer (Apt-3-F) that specifically bound to the NCL on the cell surface without being internalized into cytoplasm. With these advantages, Apt-3-F offers high value as an alternative for live-cell surface staining. Additionally, its ability to distinguish between cancer cells and normal cells makes Apt-3-F a possible tool for determining cancer prognosis and indication of RSV infections. 

## Figures and Tables

**Table 1:** Sequence information of aptamers used in this study. The linking loop region is marked as bold and underlined within the aptamer sequence.

Aptamer Names	Sequence
**Apt-1**	**GUUAGGGUUUGGGAUUG**
**Apt-2**	**GUUAGGGUUUUGGGAUUG**
**Apt-3**	**GUUAGGGUUUAUGGGAUUG**
**Apt-4**	**GUUAGGGUUUAUUGGGAUUG**
**Apt-5**	**GUUAGGGUUUAUUUGGGAUUG**
**Apt-6**	**GUUAGGGUUUAUUAUGGGAUUG**
**AS411**	**GGTGGTGGTGGTTGTGGTGGTGGTGG**

**Table 2:** Evaluation of the *CC_50_* value of Apt-3 and AS1411 against various cancer cell lines.

Cells	Apt-3 *CC_50_* (μM)	AS1411 *CC_50_* (μM)
MDA MB 468	>128	11.10
MCF7	>128	15.60
Hela	119.20	4.49

## Data Availability

Data is available to readers by directly emailing the corresponding authors.

## Supplementary Materials

The following supporting information can be downloaded at: https://www.mdpi.com/article/10.3390/ijms241814344/s1.
