# Supplementary material for: A Unique G-Quadruplex Aptamer: A Novel Approach for Cancer Cell Recognition, Cell Membrane Visualization, and RSV Infection Detection
Source: Int J Mol Sci. 2023 Sep 20;24(18):14344. doi: 10.3390/ijms241814344 (PMC10531985; doi:10.3390/ijms241814344)
Supplement: Supplementary file 1 [file ijms-24-14344-s001.zip › ijms-2599442-supplementary.pdf]

A Unique G-Quadruplex Aptamer: A Novel Approach for Cancer Cell Recognition, Cell Membrane Visualization, and RSV Infection Detection

Chao-Da Xiao [1,2,*,†], Ming-Qing Zhong [1,2,†], Yue Gao [1], Zheng-Lin Yang [1], Meng-Hao Jia [1], Xiao-Hui Hu [1], Yan Xu [3] and Xiang-Chun Shen [1,2,*]

1 State Key Laboratory of Functions and Applications of Medicinal Plants, School of Pharmaceutical Sciences, Guizhou Medical University, Guiyang 550025, China; zhongmingqing163@163.com (M.-Q.Z.); gaoyue2308@163.com (Y.G.); crazyyang168@163.com (Z.-L.Y.); jiamenghao0522@163.com (M.-H.J.); 19985602747@163.com (X.-H.H.)

2 The Key Laboratory of Optimal Utilization of Natural Medicine Resources, School of Pharmaceutical Sciences, Guizhou Medical University, Guiyang 550025, China

3 Division of Chemistry, Department of Medical Sciences, Faculty of Medicine, University of Miyazaki, Miyazaki 889-1692, Japan; xuyan@med.miyazaki-u.ac.jp

* Correspondence: xcd@gmc.edu.cn (C.-D.X.); sxc@gmc.edu.cn (X.-C.S.)

† These authors contributed equally to this work.

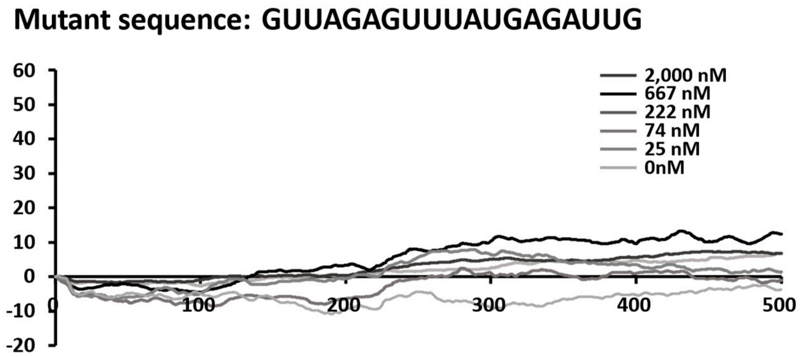

Figure S1. Sensorgrams showing mutant sequence - NCL binding (resonance units vs. time) with increasing aptamer concentration from 0 μM to 2 μM.

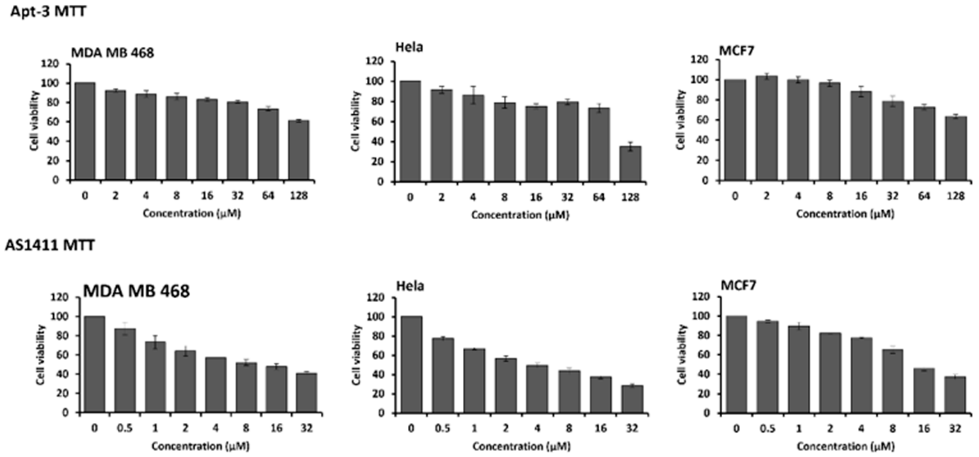

Figure S2. The cytotoxicity of Apt 3 and AS1411 was evaluated in MDA MB 468, Hela, and MCF 7 cells through an MTT assay.

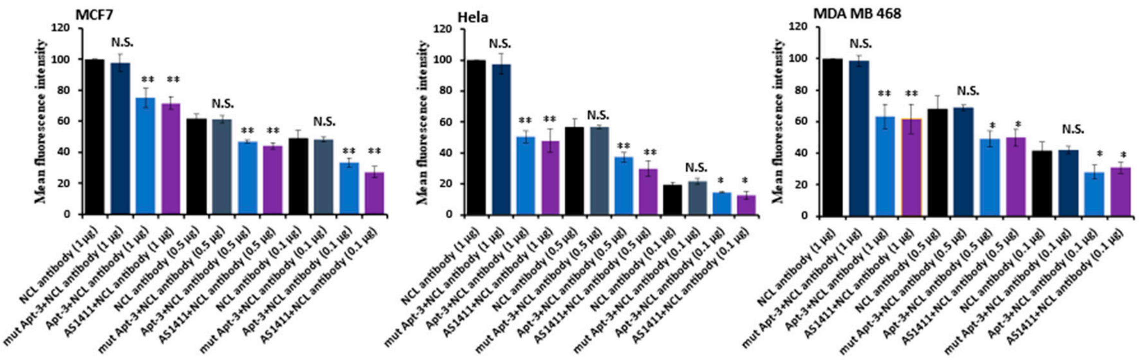

Figure S3. IHC + flow cytometry confirmed Apt-3 binding to NCL on cell membranes of MCF-7 **a**, Hela **b**, MDA MB 468 **c**. Data were expressed as the mean ± SEM (*n*=3). **p < 0.01.*p < 0.05. (Student's *t* test).

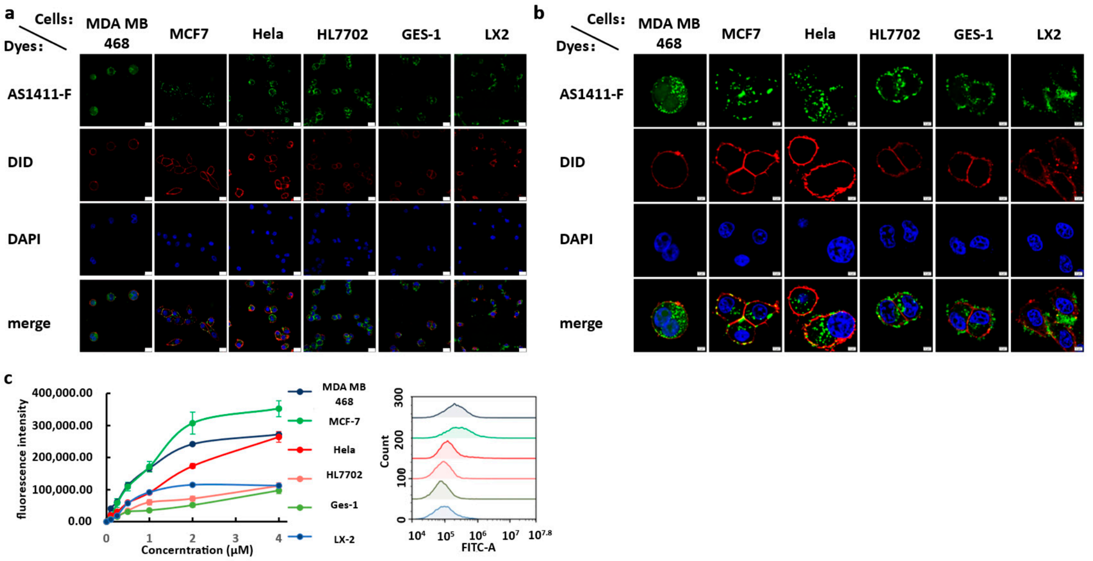

Figure S4. a Laser confocal microscope images of cancer and normal cells treated with 2 μM AS1411-F. Scale bar is 20 μm. Green channel: λ_{ex}=488 nm, λ_{em}=500–530 nm, blue channel: λ_{ex}=405 nm, λ_{em}=440-470 nm, red channel: λ_{ex}=561 nm, λ_{em}=600–700 nm. **b** High magnification images displaying the cell membrane staining of AS1411-F. Scale bar is 5 μm. **c** Left side: mean fluorescence intensity variations of different cell staining with increasing concentrations of AS1411-F detected by a flow cytometer with a fixed wavelength at λ_{ex}=488 nm, λ_{em}=530 nm. right side: The flow cytometry representation results for various cells at a 1 μM concentration of AS1411-F.

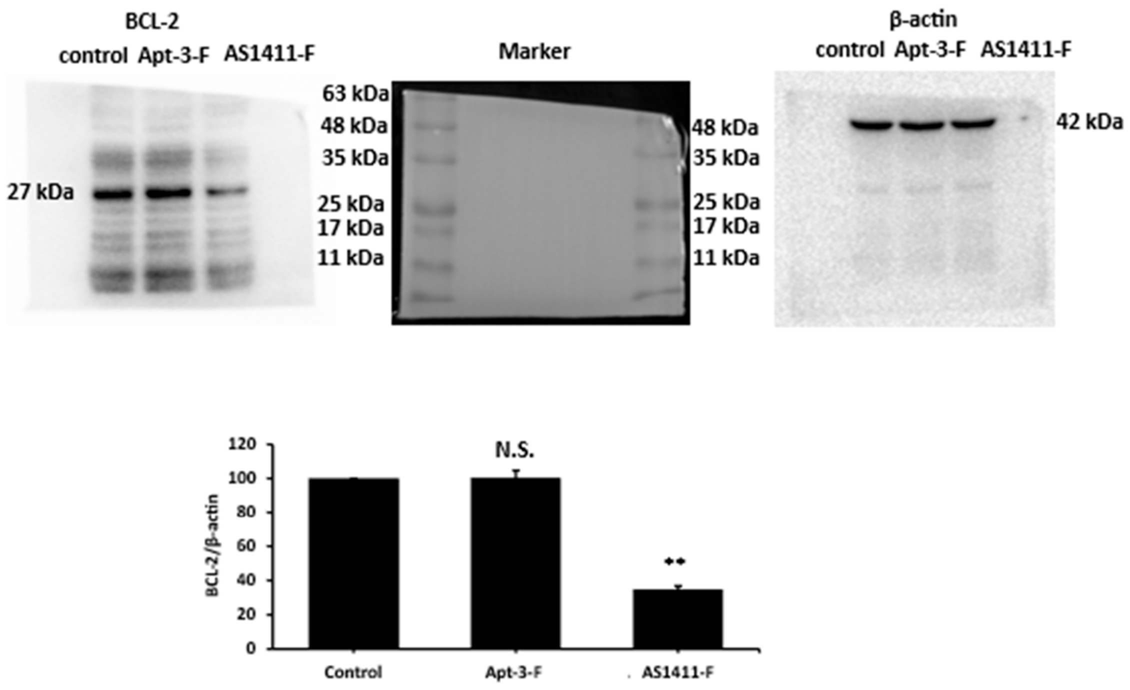

Figure S5. Western blot analysis of BCL-2 protein expression in Hela cells treated with Apt-3-F (10 μM) or AS1411(10 μM). Data were expressed as the mean ± SEM (*n*=3). **p < 0.01 (Student's *t* test). N.S. (nonsignificant) = p >0.05, vs. Ctrl group.

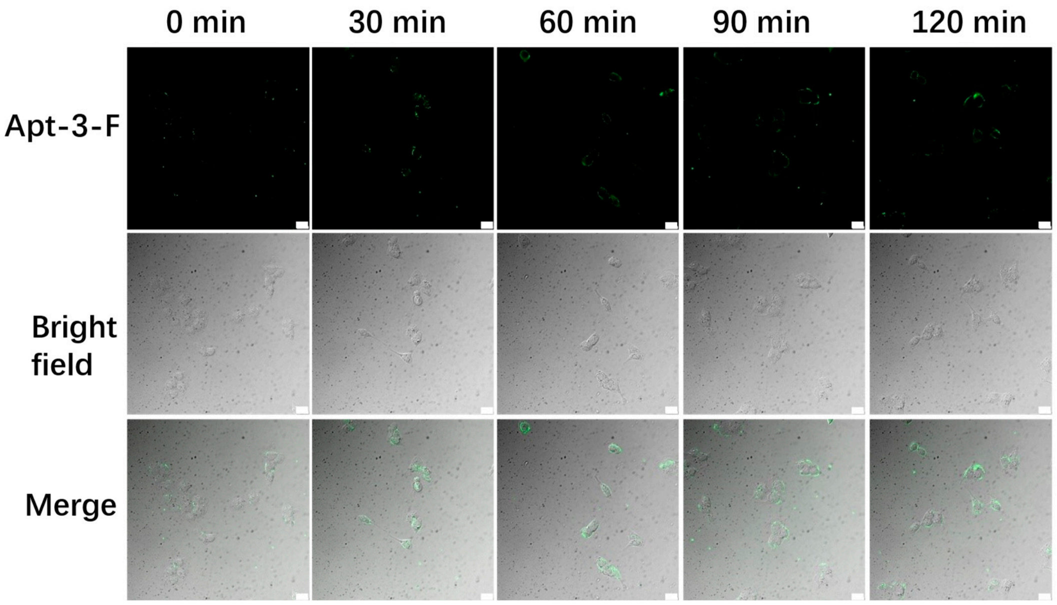

Figure S6. Apt-3-F staining images of MDCK cells treated with RSV for different time. Scale bar: 20 μm

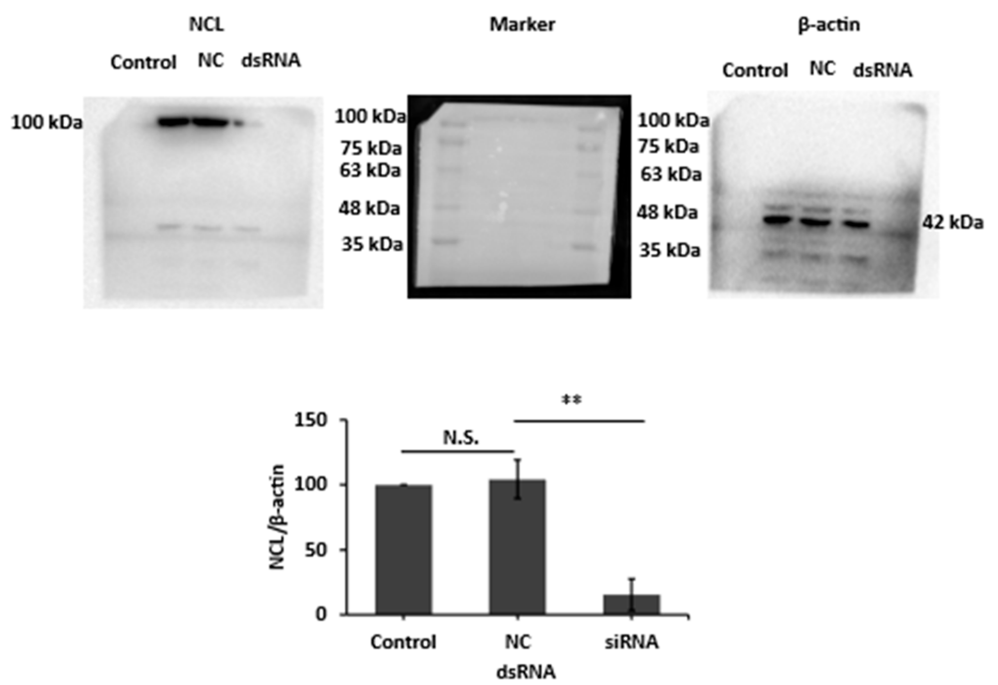

Figure S7. NCL expression was inhibited upon transfection of dsRNA into HeLa cells. The lower histograms depict the percentage of NCL repression achieved through RNAi inhibition. Data were expressed as the mean ± SEM (*n*=3). **p < 0.01 (Student's *t* test).

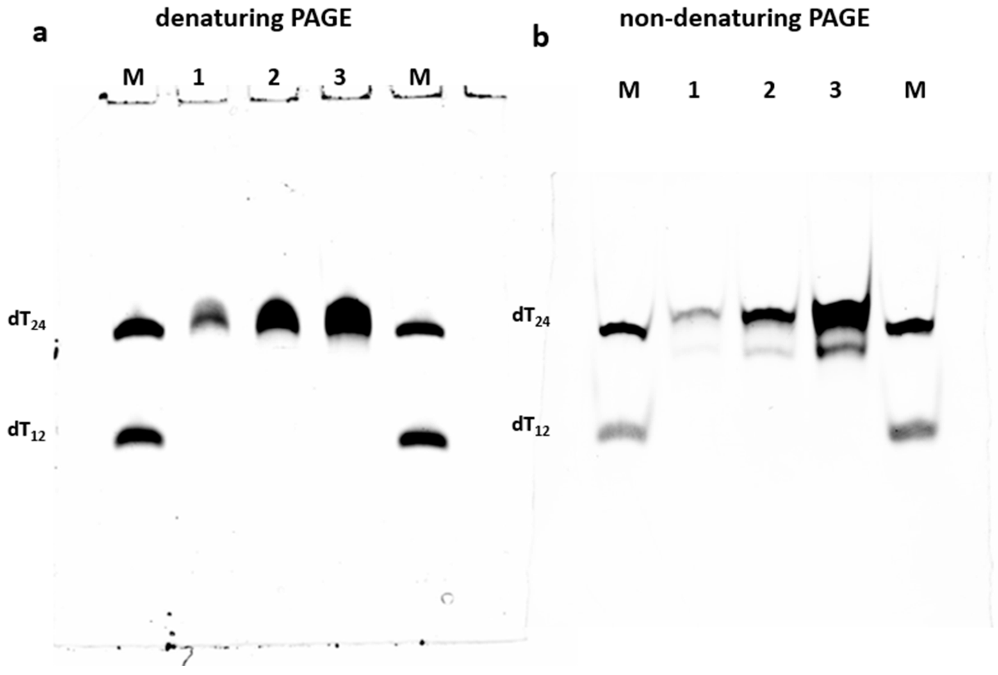

Figure S8. Denaturing and non-denaturing PAGE images of Apt-3**.** Lane M, makers dT12 and dT24; lane 1, 2, and 3, Apt-3 (100, 200 and 400 pmol).
